# Ectopic Expression of Production of Anthocyanin Pigment 1 (*PAP1*) Improves the Antioxidant and Anti-Melanogenic Properties of Ginseng (*Panax ginseng* C.A. Meyer) Hairy Roots

**DOI:** 10.3390/antiox9100922

**Published:** 2020-09-26

**Authors:** Sora Jin, Tae Kyung Hyun

**Affiliations:** Department of Industrial Plant Science and Technology, College of Agricultural, Life and Environmental Sciences, Chungbuk National University, Cheongju 28644, Korea; thfkrhehd123@naver.com

**Keywords:** anthocyanin, antioxidants, anti-melanogenic activity, ginseng hairy roots, production of anthocyanin pigment 1

## Abstract

The development of genetically engineered cell cultures has been suggested as a potential approach for the production of target compounds from medicinal plants. In this study, we generated *PAP1* (production of anthocyanin pigment 1)-overexpressing ginseng (*Panax ginseng* C.A. Meyer) hairy roots to improve the production of anthocyanins, as well as the bioactivity (e.g., antioxidant and whitening activities) of ginseng. Based on differentially expressed gene analysis, we found that ectopic expression of *PAP1* induced the expression of genes involved in the ‘phenylpropanoid biosynthesis’ (24 genes), and ‘flavonoid biosynthesis’ (17 genes) pathways, resulting in 191- to 341-fold increases in anthocyanin production compared to transgenic control (TC) hairy roots. Additionally, *PAP1*-overexpressing ginseng hairy roots exhibited an approximately seven-fold higher DPPH-free radical scavenging activity and 10-fold higher ORAC value compared to the TC. In α-melanocyte-stimulating hormone-stimulated B16F10 cells, *PAP1*-overexpressing ginseng hairy roots strongly inhibited the accumulation of melanin by 50 to 59% compared to mock-control. Furthermore, results obtained by quantitative real-time PCR, western blot, and tyrosinase inhibition assay suggested that the anti-melanogenic activity of *PAP1*-overexpressing ginseng hairy roots is mediated by tyrosinase activity inhibition. Taken together, our results suggested that the ectopic expression of *PAP1* is an effective strategy for the enhancement of anthocyanin production, which improves the biological activities of ginseng root cultures.

## 1. Introduction

Anthocyanins are water-soluble vacuolar pigments synthesized via multiple enzymes involved in the biosynthesis pathway, which is divided into the general phenylpropanoid pathway, the early flavonoid pathway, and the anthocyanin-specific pathway [1]. The most widespread anthocyanins derive from aglycone forms of anthocyanins such as cyanidin, pelargonidin, delphinidin, peonidin, petunidin, and malvidin [2]. In higher plants, anthocyanins are believed to have important functions including defense and protection against environmental stressors via reactive oxygen species (ROS)-mediated detoxification [3]. Additionally, anthocyanins have attracted attention due to their known physiological activities and their applications in the food, cosmetic, and pharmaceutical industries, as well as their many benefits to human health [4]. Therefore, anthocyanin-rich products have become increasingly popular not only due to their appealing colors but also because of their therapeutic effects.

Anthocyanin biosynthesis in plants is induced by plant hormones such as jasmonates and abscisic acid, as well as environmental factors such as light density, moisture, temperature, and ultraviolet-B radiation [5]. Chemical elicitors act as metabolite-inducing factors by mimicking stress conditions and have therefore been used to enhance anthocyanin production in plant tissues. For example, chitosan induced the expression of genes involved in the anthocyanin biosynthesis pathway, resulting in an enhanced synthesis of monomeric anthocyanins in *Vitis vinifera* grape skins [6]. Moreover, glucose treatment also increased total anthocyanin contents by 57% and 33% in China rose and Rambo radish sprouts [7]. These observations indicate that anthocyanin overproduction can be achieved by coupling cell suspension cultures with chemical induction, which can then be applied in the pharmaceutical industry. However, the low productivity in the cultured cells compared to field-grown plants coupled with their inconsistent responses to chemical elicitors limit the elicitor-induced production of secondary metabolites [8,9]. Another possible approach to improve anthocyanin production in plants is the overexpression of transcription factors known to activate anthocyanin biosynthesis genes. At the transcriptional level, anthocyanin biosynthesis is known to be tightly regulated by R2R3-MYB, MYC-like basic helix-loop-helix (bHLH), and WD40-repeat transcription factors, as well as by their coordinated interactions (MYB-bHLH-WD40 regulatory complexes) [10,11]. Among the three proteins that form the MBW complex, R2R3-MYB activates transcription of the anthocyanin biosynthetic genes via direct binding to specific DNA sequences, resulting in a sharp anthocyanin accumulation [12,13,14]. Ectopic expression of production of anthocyanin pigment 1 (*PAP1*), also known as AtMYB75, induced whole-plant anthocyanin production in dark tobacco varieties, black nightshade (*Solanum nigrum*), and *Taraxacum brevicorniculatum* [15,16,17]. Furthermore, SnMYB (*Solanum nigrum* R2R3-MYB transcription factor), SmMyb1 (*Solanum melongena* R2R3-MYB transcription factor), and the potato R2R3-MYB transcription factors (AN1, MYBA1, and MYB113) were also found to induce anthocyanin accumulation when they were transiently expressed in tobacco leaves [18,19,20], suggesting that the R2R3-MYB transcription factor is functionally exchangeable between plants and could therefore be used as a potential target for anthocyanin content enhancement using biotechnological applications and metabolic engineering.

Ginseng (*Panax ginseng* C.A. Meyer) is a popular functional and medicinal plant due to its various therapeutic effects including blood pressure control, anti-stress, anti-fatigue, anti-aging, anti-oxidative, anti-diabetic, and anti-tumor properties [21]. Our previous study investigated variations in whitening effect of ginseng berry during their ripening process and found that anthocyanins directly inhibit tyrosinase activity, which in turn inhibited α-melanocyte-stimulating hormone (α-MSH)-induced melanin production in B16F10 cells [22]. This indicates that anthocyanins are important bioactive compounds in ginseng and suggests that anthocyanins may improve the pharmaceutical properties of ginseng. Therefore, this study sought to generate *PAP1*-overexpressing transgenic ginseng hairy roots. These *PAP1*-overexpressing hairy roots were characterized using next-generation sequencing transcriptome analyses coupled with HPLC. The transgenic hairy roots were found to possess significantly higher antioxidant and anti-melanogenic activities compared with transgenic control lines, suggesting that overexpressing *PAP1* can be an easy and cost-effective approach to improve the therapeutic value of plants used in the cosmetic and pharmaceutical industries.

## 2. Materials and Methods

### 2.1. Plant Materials and Growth Conditions

After removing seed coat, the ginseng (Korean ginseng, *Panax ginseng* C.A. Meyer) seeds were sterilized as described by Woo et al. [23]. Afterward, zygotic embryos were surgically separated from the sterilized seeds, transferred to a solidified 1/2 MS medium with 3% sucrose and 0.25% gelrite, and cultured in a growth chamber with a long photoperiod (16 h light/8 h dark) at 22 °C. Cotyledons and hypocotyls from 3-week-old in vitro cultured ginseng seedlings were then used for the induction of hairy roots.

### 2.2. Generation of PAP1-Overexpressing Ginseng Hairy Roots

PAP1/pK7WGF2 construct kindly provided by Chhon et al. [17] was transformed into *Agrobacterium rhizogenes* R1601. *PAP1*-overexpressing ginseng hairy roots were generated as described by Woo et al. [23] and were then selected with antibiotic-containing (50 mg/L of kanamycin and 250 mg/L of cefotaxime) B5 medium with 3% sucrose and 0.25% gelrite, followed by reverse transcriptase-PCR (RT-PCR) to identify the anthocyanin accumulation phenotype. The selected hairy roots were then cultured on gelrite-solidified B5 medium at 22 °C in the dark, and sub-cultured every 4-weeks. In addition, we selected hairy root lines, which were survived on selection medium, but exhibited no expression of *PAP1* and no accumulation of anthocyanins. These hairy root lines were defined as transgenic controls (TCs).

### 2.3. Transcriptome Analysis

Total RNA from each sample was extracted using the FavorPrep Plant Total RNA Mini Kit (Favorgen Biotech Corp, Pingtung, Taiwan) and quantified using a DeNovix DS-11 spectrophotometer (DeNovix Inc., Wilmington, DE, USA). After synthesizing cDNA, the cDNA library was sequenced on an Illumina HiSeq™ 2500 system using the *Panax ginseng* genome version 1 as reference (http://ginsengdb.snu.ac.kr/data.php). All reads were deposited in the National Agricultural Biotechnology Information Center (NABIC, http://nabic.rda.go.kr). Differentially expressed genes (DEGs) between the transgenic lines and the TC line were identified with the DEseq bioinformatics package. For functional analysis, gene ontology (GO) enrichment, Kyoto Encyclopedia of Genes and Genomes (KEGG) pathway enrichment, and MapMan metabolism overview maps of DEGs were conducted as described by Eom et al. [24]. A corrected *p*  ≤  0.05 was established as the significance threshold.

### 2.4. Quantitative Real-Time PCR (qRT-PCR) Analysis

To confirm the expression profiles observed in the RNA-Seq data, total RNA from each hairy root line was extracted using the FavorPrep Plant Total RNA Mini Kit. Additionally, total RNA from B16F10 melanoma cells was extracted using the TRIzol reagent (Molecular Research Center, Cincinnati, OH, USA). cDNA was synthesized using ReverTra Ace™ qPCR RT Master Mix with gDNA Remover (TOYOBO, Co., Ltd., Osaka, Japan). qRT-PCR was performed using CFX96TM Real-time system (Bio-Rad, Hercules, CA, USA). The relative expression level of each gene was normalized to the expression of actin (i.e., an internal reference gene). Table S1 lists the primer pairs used for qRT-PCR.

### 2.5. Sample Extraction and Analysis of Total Anthocyanin (TAC) and Ginsenosides Contents

First, 4-week-old *PAP1*-overexpressing ginseng hairy root lines (R10 and R12) and TC were harvested and freeze-dried, after which 70% ethanol extracts were prepared as described by Jin et al. [22].

TAC content was determined as described by Choi et al. [25]. Briefly, absorbances were measured at 530 nm (A530) and 657 nm (A657) using microplate spectrophotometer (BioTek Instruments, Inc., Winooski, VT, USA), after which TAC was calculated with the following formula: TAC = (A530 – 0.25 × A657) / mg of dry weight

The composition of ginsenosides in 70% EtOH extracts was determined with an Agilent Technologies 1200 series HPLC (Conquer Scientific, San Diego, CA, USA) coupled with Kinetex 2.6u XB-C18 100A column (100 × 4.6 mm). The mobile phase consisted of acetonitrile (solvent A) and water (mobile phase B), and the system was operated with a gradient elution program as described by Jin et al. [22]. The results were quantified using a Ginseng Ginsenosides Mix (Sigma-Aldrich Co., St. Louis, MO, USA) as standard.

### 2.6. Determination of Antioxidant Capacity

The 1,1-diphenyl-2-picrylhydrazyl (DPPH) radical scavenging activity was determined as described by Jin et al. [22]. The concentration of sample required for 50% reduction of DPPH absorbance (RC_50_) was then calculated, and butylated hydroxytoluene (BHT) was used as a positive control for the antioxidant assay.

Moreover, the ferric reducing antioxidant power (i.e., the ability to reduce Fe^3+^ to Fe^2+^) was estimated as described by Jin et al. [22]. Various volumes (10 µL, 20 µL and 30 µL) of each samples (10 mg/mL) were mixed with 200 µL sodium phosphate buffer (0.2 M, pH 6.6) and 200 µL potassium ferricyanide (1%, *w/v*). After stopping the reaction by adding 1 mL of 10% trichloroacetic acid, 0.5 mL of the reaction mixture was mixed with an equal volume of distilled water and 0.1 mL of 0.1% (*w/v*) ferric chloride. The absorbance was measured at 750 nm.

The oxygen radical absorbance capacity (ORAC) assay was used to analyze the antioxidant potential of each sample, as described by Jin et al. [22]. Each sample (25 µL) was mixed with 150 µL of 0.08 µM fluorescein and 25 µL of 75 mM phosphate buffer (pH 7.0) and then incubated at 37 °C for 10 min. Afterward, the fluorescence intensity was measured every minute for 90 min at 37 °C using a Synergy^TM^ HTX Multi-mode Microplate Reader (BioTek) with fluorescent filters (485 nm excitation and 530 nm emission). The ORAC value of each sample was calculated based on a Trolox standard curve (TE).

### 2.7. Melanin Content and Cell Viability Analyses

B16F10 melanoma cells were cultured in Dulbecco’s modified Eagle’s medium (DMEM) supplemented with 10% fetal bovine serum, 100 U/mL penicillin and 100 μg/mL streptomycin in an incubator containing 5% CO_2_ at 37 °C.

The inhibitory effect of each sample on α-MSH-induced melanin contents in B16F10 cells was analyzed as described by Jin et al. [22]. The B16F10 cells were exposed to 50 µg/mL of each sample or DMSO (as a mock control) with 50 nM α-MSH for 48 h. After harvesting the cells, the cell pellets were solubilized in 1 N NaOH containing 10% DMSO at 65 °C for 1 h, after which the absorbance was measured at 490 nm. The melanin contents were expressed as percentages of the mock control. Furthermore, the viability of α-MSH-stimulated B16F10 cells treated with each sample or DMSO was determined via the MTT assay [22].

The in vitro inhibitory effects of each sample against tyrosinase activity were determined using the Tyrosinase Inhibitor Screening Kit (BioVision, Milpitas, CA, USA).

### 2.8. Immunoblotting Analyses

Proteins were extracted from α-MSH-stimulated B16F10 cells treated with each sample or DMSO using RIPA lysis buffer (50 mM Tris-HCl pH 7.5, 150 mM NaCl, 1% Triton X-100, 0.1% SDS, 0.5% sodium deoxycholate, 1 mM EDTA, and 10 mM NaF), and quantified using the Pierce™ BCA Protein Assay Kit (Thermo Scientific™, Rockford, IL, USA). The proteins were separated using SDS-page gels and transferred to polyvinylidene difluoride (PVDF) membranes. The membranes were then incubated with anti-tyrosinase antibody or anti-actin antibody and the immune complexes were visualized with the ECL reagent using an Azure c280 imaging system (Azure Biosystems, Inc., Dublin, CA, USA).

### 2.9. Statistical Analysis

All experiments were independently conducted three times and the results are reported herein as the mean ± standard error (SE). ANOVA analysis was performed using SPSS (Version 23, IBM, Armonk, NY, USA), and significant differences between groups were identified using Duncan’s Multiple Range Test (*p* < 0.05).

## 3. Results and Discussion

### 3.1. Characterization of PAP1-Overexpressing Ginseng Hairy Roots

**Figure 1:**
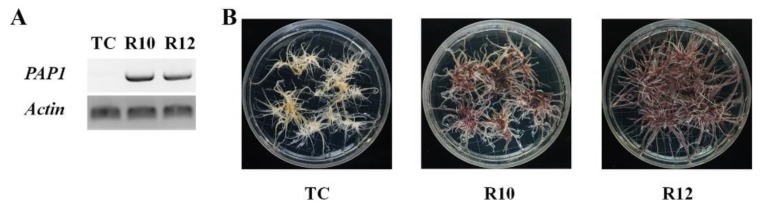
Identification and characterization of *PAP1*-overexpressing transgenic lines. (**A**) *PAP1* expression in transgenic ginseng hairy roots was confirmed via RT-PCR. (**B**) Phenotyping of *PAP1*-overexpressing ginseng hairy roots. Two transgenic lines (R10 and R12) and a transgenic control (TC) were selected for further studies.

In addition to their well-characterized roles in plant physiological processes, dietary intake of anthocyanins has also been linked to human health benefits. Therefore, to improve the pharmaceutical properties of ginseng, our study sought to generate an anthocyanin overproducing ginseng strain via ectopic over-expression of *PAP1* coupled with *A*. *rhizogenes*-mediated hairy root production. By co-cultivating ginseng seedling cotyledons or hypocotyls with appropriate *Agrobacterium* cultures and selecting them for *PAP1* expression (Figure 1A) with an antibiotic medium, several transgenic lines were found to exhibit anthocyanin-accumulating phenotypes (Figure 1B), of which two (R10 and R12) were selected for further studies. 

**Figure 2:**
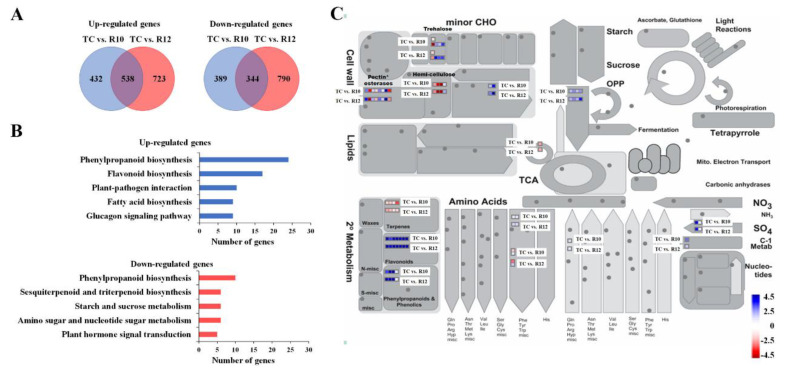
Analysis of differentially expressed genes (DEGs) induced by *PAP1* overexpression. (**A**) Venn diagram of DEGs (TC vs R10 and TC vs R12). KEGG pathway enrichment analysis (**B**) and MapMan metabolism overview (**C**) using 882 DEGs, which were up- or down-regulated DEG in both *PAP1*-overexpressing hairy root lines. The different colors represent the log2 values of expression levels of DEGs. Transgenic lines: R10 and R12; Transgenic control: TC.

Based on the number of MYB conserved domain repeats, the MYB transcription factors in plants are divided into four groups: MYB-related (MYBR, which only contains one R1- or R2-like repeat), R2R3-MYB (containing two R2/3R-like repeats), 3R-MYB (containing three R1/R2/R3-like repeats), and atypical MYB (4R-MYB, four R1/R2-like repeats; CDC5-like) [26]. Among these, the R2R3-MYB is the largest group and is the main regulator of the structural genes involved in the anthocyanin biosynthetic pathway [27] but is also known to regulate other processes including cell cycle control, meristem formation, cellular morphogenesis, hormone signaling, secondary metabolism, and abiotic and biotic stress responses [26,28,29]. To investigate the effects of *PAP1* over-expression on the gene expression of ginseng hairy roots, cDNA libraries from the TC, R10, and R12 lines were constructed and sequenced via the Illumina HiSeq™ 2500 sequencing platform. After removing low-quality reads, 45.8 to 48.6 million clear reads (6.64 to 7.02 Gb) were acquired from each sample for further DEG analysis (Table S2). Two pair-wise comparisons were conducted (TC vs. R10 and TC vs. R12) to identify PAP1-induced DEGs. As shown in Figure 2A, a total of 1,694 DEGs (961 up-regulated and 733 down-regulated) were detected by comparing the TC and R10 libraries. Moreover, 1,261 genes were found to be up-regulated and 1,134 genes were downregulated in R12. Inter-individual variations of transgene expression in nuclear-transformed plants are mediated by several factors including transgene copy numbers, RNA silencing, transgene insertion site, and promoter and terminator suitability [30], suggesting that line-specific DEGs might result from inter-individual variations. Therefore, 882 DEGs (538 up-regulated DEGs and 344 down-regulated DEGs in both *PAP1*-overexpressing hairy root lines) were selected for further studies. 

KEGG pathway enrichment is a systematic analysis that enables the identification of gene groups with related functions, and this pathway enrichment analysis approach is a useful tool to better understand the biological functions of genes [31]. To investigate which DEGs were activated and suppressed in different pathways, DEGs were annotated with corresponding Enzyme Commission (EC) numbers using BLASTx and mapped to their corresponding KEGG pathways. As shown in Figure 2B, the results of the KEGG pathway enrichment analysis revealed that upregulated DEGs were mainly enriched in the ‘phenylpropanoid biosynthesis’ (24 genes), and ‘flavonoid biosynthesis’ (17 genes) pathways, whereas downregulated DEGs were mainly associated with the ‘phenylpropanoid biosynthesis’ (10 genes) and ‘sesquiterpenoid and triterpenoid’ (6 genes) pathways. Interestingly, PAP1 overexpression led to both increases and decreases in the expression of phenylpropanoid biosynthesis-related genes. As shown in Table S3, genes encoding proteins containing the berberine and berberine-like domain (PF08031) or the peroxidase domain (PF00141) were up- or down-regulated by PAP1, indicating that the functional redundancy of these gene families might be required for homeostatic responses. Furthermore, MapMan analysis was performed to visualize the metabolic pathways associated with the identified PAP1-induced DEGs. The MapMan enrichment results indicated that the DEGs were mainly involved in trehalose, hemicellulose, pectin*esterases, terpenes, flavonoids, and phenylpropanoids (Figure 2C). In *Arabidopsis*, up-regulation of cellulose synthase isoforms (CesA4, 7, and 8) involved in the biosynthesis of secondary cell wall cellulose microfibrils was observed in *myb75-1* (loss-of-function of *pap1* mutant) plant stems [32]. In addition, eight pectin methylesterases involved in cell wall remodeling and mucilage release [33] were found to be PAP1-induced DEGs (Figure 2C). PAP1 regulates secondary cell wall deposition and mucilage formation via interacting KNAT7 transcription factor in *Arabidopsis* [32], suggesting that PAP1 might be involved in the secondary cell wall formation via controlling the pectin methylesterases, as well as cellulose synthases. The treatment of trehalose causes the accumulation of anthocyanins [34]. In *PAP1*-overexpressing ginseng hairy roots, three trehalose 6-phosphate phosphatases, which are required for the production of free trehalose [34], were up-regulated (Figure 2C), indicating that PAP1 plays a role in sugar-signaling involved in anthocyanin accumulation. Taken together, these indicate that PAP1 has a similar function as a transcription factor in both heterologous and homologous systems.

### 3.2. PAP1 Overexpression Enhanced Anthocyanin Production but Reduced Ginsenoside Production in Ginseng Hairy Roots

**Figure 3:**
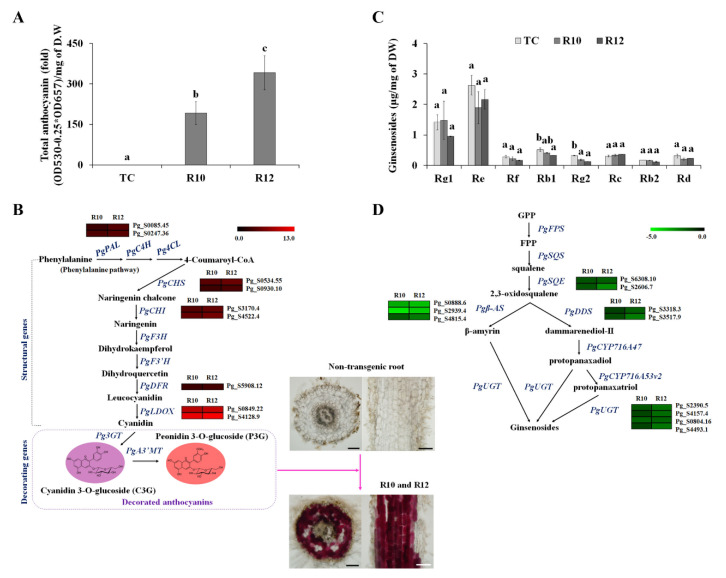
Effect of PAP1 on the anthocyanin and ginsenoside biosynthetic pathways. Anthocyanin contents (**A**) and PAP1-induced DEGs involved in the anthocyanin biosynthetic pathway (**B**). Sections obtained from *PAP1*-overexpressing hairy roots exhibited anthocyanin accumulation. Alteration of ginsenoside contents (**C**) and PAP1-induced DEGs involved in the ginsenoside biosynthetic pathway (**D**). Expression (**B** and **D**) is indicated as a log2 ratio of *PAP1*-overexpressing hairy roots relative to the TC and visualized in heatmaps. Values with the same letter were deemed not significantly different (*p* < 0.05). Scale bar = 200 µm. Transgenic lines: R10 and R12; Transgenic control: TC.

PAP1 is a known positive regulator of genes involved in the phenylpropanoid pathway [35]. In *Arabidopsis*, *PAP1* overexpression leads to the up-regulation of dihydroflavonol-4-reductase (*DFR*) and leucoanthocyanidin dioxygenase (*LDOX*). Moreover, elevated levels of phenylalanine ammonia-lyase (*PAL*), cinnamic acid-4-hydroxylase (*C4H*), flavanone 3-hydroxylase (*F3H*), and *DFR* transcription were observed in *PAP1*-overexpressing petunia flowers [36]. *PAP1*-overexpressing ginseng hairy roots exhibited 191- to 341-fold increases in anthocyanin levels compared to TC (Figure 3A), and mRNA levels of ginseng *PALs*, chalcone synthases (*CHSs*), chalcone isomerases (*CHIs*), *DFRs*, and *LDOXs* were elevated by PAP1 (Figure 3B and Figure S1). Although flavonoids and terpenoids are derived from distinct metabolic pathways, it has been shown that PAP1 enhances the production of phenylpropanoid and terpenoid scent compounds in rose flowers [37]. This indicates that the MYB family plays an important role in controlling metabolic activity between the flavonoid and terpenoid biosynthetic pathways [37,38]. Particularly, ginsenosides, steroids, and triterpenoid glycosides produced from terpenoid precursors [39] are known to be major active constituents of ginseng. To investigate the effect of PAP1 on the production of ginsenosides, we analyzed the composition of ginsenosides and the transcription levels of genes involved in the ginsenoside biosynthesis pathway. As shown in Figure 3C, low levels of ginsenoside Rb1 and Rg2 were observed in *PAP1*-overexpressing lines compared with TC. Additionally, ginseng squalene epoxidases (*PgSQEs*), dammarenediol II synthases (*PgDDSs*), β-amyrin synthases (*PgBASs*), and UDP-glycosyltransferase (*PgUGTs*) were identified as DEGs (Figure 2B and Figure 3D), and the expression patterns of these genes were validated using qRT-PCR (Figure S2), thereby confirming that the down-regulation of these genes affected the production of some ginsenosides including Rb1 and Rg2. It has been shown that bHLH and WRKY transcription factors are positive regulators in the biosynthesis of triterpenoid saponins [40,41,42]. In *Medicago truncatula* hairy roots, jasmonate-inducible subclade Iva bHLH transcription factors increased the transcription level of genes involved in the mevalonate precursor and saponin-specific pathways, resulting in the accumulation of saponins [40]. 

Furthermore, PgMYB2 (*P. ginseng* MYB2), a homolog of AtMYB6 (*Arabidopsis* R2R3-MYB transcription factor), has been suggested to be a major factor for the enhancement of ginsenoside production via the induction of *PgDDS* transcription [43]. These results indicate that the production of ginsenosides is likely controlled by several transcription factors, and the reduction of some ginsenoside levels by PAP1-overexpression might be attributed to secondary metabolite imbalances.

### 3.3. PAP1 Improved the Antioxidant Properties of Ginseng Hairy Roots

**Figure 4:**
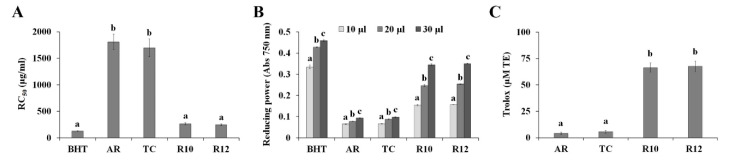
Antioxidant activities of *PAP1*-overexpressing ginseng hairy roots were measured by DPPH-free radical scavenging (**A**), reducing power (**B**), and ORAC (**C**) assays. DPPH-radical scavenging activity was calculated as RC_50_. The ORAC values of each extract are expressed as μM of Trolox equivalents. The bars represent the mean ± SE of three independent experiments. Values in the same column with different superscripted letters are significantly different (*p* < 0.05). Transgenic lines: R10 and R12; Transgenic control: TC; Ginseng adventitious root: AR.

Phytochemicals are known to possess antioxidant properties, and can therefore be used for medicinal purposes. Particularly, anthocyanins were recently identified as promising therapeutic agents against reactive oxygen species (ROS)-triggered diseases [44]. Therefore, our study also investigated the effect of *PAP1*-overexpression on the antioxidant properties of ginseng hairy roots. As shown in Figure 4A, R10 (RC_50_ = 264.3 μg/mL) and R12 (RC_50_ = 246.7 μg/mL) exhibited strong DPPH-free radical scavenging activity compared to ginseng adventitious roots (AR, RC_50_ = 1807.2 μg/mL) and TC (RC_50_ = 1697.7 μg/mL). Moreover, 30 µL of 70% EtOH extracts (10 mg/mL) of *PAP1*-overexpressing hairy roots exhibited OD700 values ranging from 0.34 to 0.35 and stronger activity than that of AR (0.093) or TC (0.098) (Figure 4B). Antioxidant activity is mediated by various mechanisms including hydrogen atom transfer (HAT) and single electron transfer (SET). The DPPH-free radical scavenging assay is based on a mixture (SET/HAT) of these mechanisms, whereas reduction capacity assay is exclusively SET-based [45]. To further characterize the antioxidant properties of 70% EtOH extracts of *PAP1*-overexpressing hairy roots, we analyzed their antioxidant capacities using the ORAC assay, which is a HAT-based assay. R10 and R12 at 30 µg/mL exhibited ORAC values of 66.4 and 67.6 µM TE, respectively, and TC at 30 µg/mL had an ORAC value of 5.6 µM TE (Figure 4C), suggesting that *PAP1*-overexpression increased the occurrence of HAT- and SET-capable antioxidant molecules. The antioxidant activity of anthocyanins has been demonstrated to involve both HAT and SET mechanisms [46]. Taken together, our results indicate that the accumulation of anthocyanins in ginseng hairy roots resulted in enhanced antioxidant properties, although the extracts of R10 and R12 exhibited lower levels of antioxidant activity relative to the positive BHT control.

### 3.4. Ectopic PAP1 Expression Increased the Anti-Melanogenic Potential of Ginseng Hairy Roots

**Figure 5:**
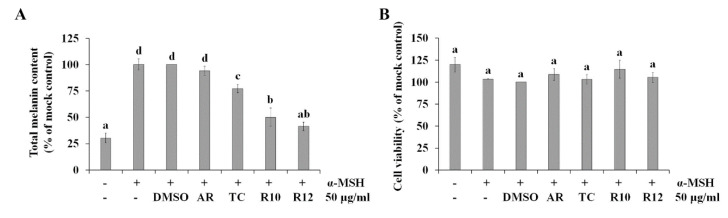
Anti-melanogenic activity of *PAP1*-overexpressing ginseng hairy root extracts. The effect of *PAP1*-overexpressing ginseng hairy root extracts on melanin production (**A**) and cell viability (**B**) was analyzed in α-MSH-stimulated B16F10 cells. The values represent the means of three replicates ± SE. Values with the same letter were deemed not significantly different by Duncan’s multiple range tests. Transgenic lines: R10 and R12; Transgenic control: TC; Ginseng adventitious root: AR.

We previously demonstrated that the anti-melanogenic activity of ginseng berries was mediated by the presence of anthocyanins, which can interact with copper-coordinating histidines and second shell residues in tyrosinase [22]. Therefore, we hypothesized that *PAP1* overexpression could also improve the anti-melanogenic potential of ginseng hairy roots. To assess this, the inhibitory effects of 70% EtOH extracts on α-MSH-induced melanin accumulation in B16F10 melanoma cells were analyzed. As illustrated in Figure 5A, 50 μg/mL R10 and R12 extracts significantly inhibited melanin production in α-MSH-stimulated B16F10 cells, whereas AR and TC had little or no effect on α-MSH-induced melanin production. Specifically, R10 and R12 decreased melanin content in α-MSH-stimulated cells by 50% and 59%, respectively. Additionally, no significant cytotoxic effects were observed on α-MSH-stimulated B16F10 cells (Figure 5B), indicating that the anti-melanogenic activity of R10 and R12 is not mediated by cytotoxicity. 

**Figure 6:**
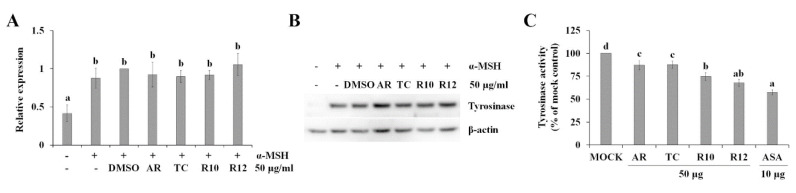
Effect of PAP1-overexpressing ginseng hairy root extracts on the transcription (**A**), translation (**B**), and activity of tyrosinase (**C**). The expression of tyrosinase was normalized to that of β-actin (**A**). (**B**) Total proteins were extracted and assayed by western blotting using tyrosinase-specific antibody. The inhibitory effect of *PAP1*-overexpressing ginseng hairy root extracts on tyrosinase activity was analyzed in cell-free conditions, as described in the Materials and Methods. The values represent the means of three replicates ± SE. Values with different superscripted letters are significantly different (*p* < 0.05). Transgenic lines: R10 and R12; Transgenic control: TC; Ginseng adventitious root: AR.

Tyrosinase is a rate-limiting enzyme that catalyzes the synthesis of the pigment melanin from tyrosine [47]. Therefore, melanin production is mainly controlled by the transcription and activation of tyrosinase in melanoma cells [48]. However, the anti-melanogenic activities of R10 and R12 were not modulated by the inhibition of α-MSH-induced transcription (Figure 6A) and translation (Figure 6B) of tyrosinase. In cell-free conditions, the residual tyrosinase activity was 74.7% and 67.7% of that of the mock control in the 50 μg/mL R10 and R12 treatments, respectively (Figure 6C), indicating that R10 and R12 directly inhibited tyrosinase activity, resulting in the inhibition of α-MSH-induced melanin production in B16F10 melanoma cells. These results suggested that the accumulation of anthocyanins by overexpression of *PAP1* improved the anti-melanogenic activity of ginseng hairy roots through the inhibition of tyrosinase activity.

## 4. Conclusions

In this study, *PAP1*-overexpressing ginseng hairy roots were generated to improve their biological activities, including their antioxidant and anti-melanogenic properties. Our results suggested that enhancing anthocyanin production is a promising strategy to improve the therapeutic value of ginseng. Although the safety of genetically modified organisms for human consumption must still be addressed, we hope that our results will encourage future research on the improvement of secondary metabolite production based on genetic engineering to enhance the pharmaceutical value of medicinal plants.

## Supplementary Materials

The following are available online at https://www.mdpi.com/2076-3921/9/10/922/s1, Figure S1. The expression patterns of the selected genes were analyzed using qRT-PCR. Table S1. Primer sequences for PCR analysis. Table S2. Summary of RNA sequencing data from three RNA libraries. Table S3. DEGs involved in phenylpropanoid biosynthesis pathway.
