# Supplementary material for: Ectopic Expression of Production of Anthocyanin Pigment 1 (PAP1) Improves the Antioxidant and Anti-Melanogenic Properties of Ginseng (Panax ginseng C.A. Meyer) Hairy Roots
Source: Antioxidants (Basel). 2020 Sep 26;9(10):922. doi: 10.3390/antiox9100922 (PMC7601150; doi:10.3390/antiox9100922)
Supplement: Supplementary file 1 [file antioxidants-09-00922-s001.pdf]

Supplementary Materials:

Table S1. Primer sequences for PCR analysis.

	Source	Primer	Sequences (5`-3`)	Accession number
RT-RCR	*Arabidopsis thaliana*	PAP1-F	GGAGGGTTCGTCCAAAG	AT1G56650
		PAP1-Rev	TCAAATTTCACAGTCTC	
qRT-PCR	*Panax ginseng*	Pg_S3170.4-F	GATTCCGCCGTGGGATCACT	Pg_S3170.4
		Pg_S3170.4-Rev	ACGAACCCGTAACCACATCCC	
		Pg_S4522.4-F	ACCTTTGCATAGACCTGGCA	Pg_S4522.4
		Pg_S4522.4-Rev	AAATACACAGGCCACTTGGG	
		PgF3'H-F	CAGGAGCGAGTGAAATTGGG	Pg_S6057.8
		PgF3'H -Rev	GGCTTTAGGATCGACAGCAC	
		Pg_S5908.12-F	AAATGGTTCGAGTGGTGCTG	Pg_S5908.12
		Pg_S5908.12-Rev	ATGGCAACCCTCTATGGCTT	
		Pg_S0849.22/Pg_S4128.9-F	TTGTGGCCAACTTGAATGGG	Pg_S0849.22
		Pg_S0849.22/Pg_S4128.9-Rev	GGCCTCTTAGCTCCTTAGCA	Pg_S4128.9
		Pg_S6308.10-F	AAGCTAGCGCTCTGCTCATT	Pg_S6308.10
		Pg_S6308.10-Rev	TGATGACGTCCGTACTTCCG	
		Pg_S3318.3-F	ATATATCAGCGGAACGATTGACAC	Pg_S3318.3
		Pg_S3318.3-Rev	ATTCAGCTAATCCTTCTCCTAGCAA	
		PgActin-F	CCCGAGAGAAAGTATAGTGTATGGA	KU215665.1
		PgActin-Rev	TAGAGCTCTTCAACAACCACTTTTT	
qRT-PCR	B16F10 cell	Tyrosinase-F	ATAGGTGCATTGGCTTCTGG	NM_011661.5
		Tyrosinase-Rev	CCAACGATCCCATTTTTCTT	
		β-actin-F	CCCACTCCTAAGAGGAGGATG	NM_007393.5
		β -actin-Rev	AGGGAGACCAAAGCCTTCAT	

Table S2. Summary of RNA sequencing data from three RNA libraries.

Sample No.	Clean reads	Clean bases (Gb)	Accession number (NABIC)
TC	48,627,260	7.02	NN-6324
R10	45,853,294	6.63	NN-6607
R12	45,838,450	6.43	NN-6608

Table S3. DEGs involved in phenylpropanoid biosynthesis pathway.

DEGs	Gene ID	Description	Pfam accession
Up-regulated DEGs	Pg_S0085.45	Aromatic amino acid lyase	PF00221
	Pg_S0213.76	Alpha/beta hydrolase family	PF12697
	Pg_S0247.35	Aromatic amino acid lyase	PF00221
	Pg_S0247.36	Aromatic amino acid lyase	PF00221
	Pg_S1135.17	Aromatic amino acid lyase	PF00221
	Pg_S1239.2	Aromatic amino acid lyase	PF00221
	Pg_S1430.2	Glycosyl hydrolase family 3 N terminal domain	PF00933
	Pg_S1660.14	Berberine and berberine like	PF08031
	Pg_S1951.9	Peroxidase	PF00141
	Pg_S2677.23	Peroxidase	PF00141
	Pg_S2748.8	Peroxidase	PF00141
	Pg_S2937.4	Berberine and berberine like	PF08031
	Pg_S3049.7	Peroxidase	PF00141
	Pg_S3049.8	Peroxidase	PF00141
	Pg_S3071.14	Alpha/beta hydrolase family	PF12697
	Pg_S3293.3	Glycosyl hydrolase family 1	PF00232
	Pg_S3787.4	Berberine and berberine like	PF08031
	Pg_S5131.11	Peroxidase	PF00141
	Pg_S5463.5	Peroxidase	PF00141
	Pg_S5463.6	Peroxidase	PF00141
	Pg_S5731.2	O-methyltransferase	PF01596
	Pg_S6389.7	AMP-binding enzyme C-terminal domain	PF13193
	Pg_S6389.8	AMP-binding enzyme	PF00501
	Pg_S6458.1	Peroxidase	PF00141
Down-regulated DEGs	Pg_S0245.11	Berberine and berberine like	PF08031
	Pg_S0614.2	Transferase family	PF02458
	Pg_S0616.15	FAD binding domain	PF01565
	Pg_S1036.5	Berberine and berberine like	PF08031
	Pg_S1212.23	Glycosyl hydrolase family 1	PF00232
	Pg_S1271.11	Peroxidase	PF00141
	Pg_S1410.4	UDP-glucoronosyl and UDP-glucosyl transferase	PF00201
	Pg_S2096.11	Transferase family	PF02458
	Pg_S3247.4	Berberine and berberine like	PF08031
	Pg_S3641.5	Peroxidase	PF00141

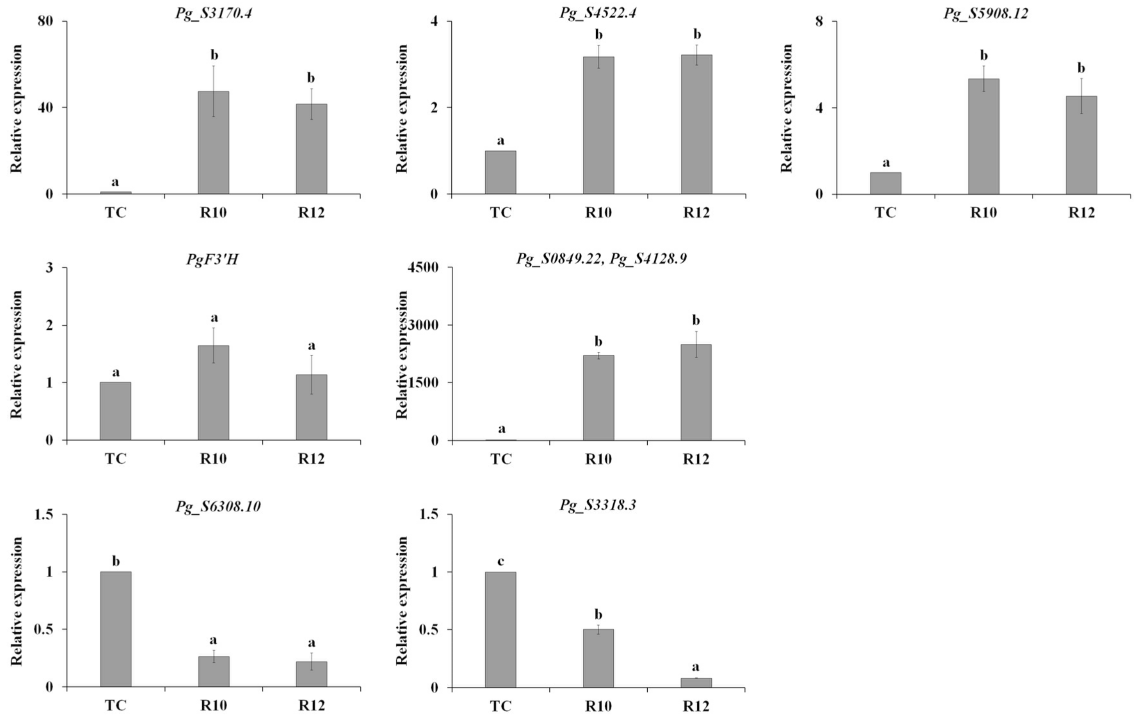

Figure S1. The expression patterns of the selected genes were analyzed using qRT-PCR.
